# Molecular Characterization of the Na^+^/H^+^-Antiporter NhaA from *Salmonella* Typhimurium

**DOI:** 10.1371/journal.pone.0101575

**Published:** 2014-07-10

**Authors:** Christopher J. Lentes, Syed H. Mir, Marc Boehm, Constanta Ganea, Klaus Fendler, Carola Hunte

**Affiliations:** 1 Institute for Biochemistry and Molecular Biology, ZBMZ, BIOSS Centre for Biological Signalling Studies, University of Freiburg, Freiburg, Germany; 2 Faculty of Biology, University of Freiburg, Freiburg, Germany; 3 Department of Clinical Biochemistry, University of Kashmir, Srinagar, India; 4 Department of Molecular Membrane Biology, Max Planck Institute of Biophysics, Frankfurt am Main, Germany; 5 Biophysical Department, Faculty of Medicine, Carol Davila University of Medicine and Pharmacy, Bucharest, Romania; 6 Department of Biophysical Chemistry, Max Planck Institute of Biophysics, Frankfurt am Main, Germany; Indian Institute of Science, India

## Abstract

Na^+^/H^+^ antiporters are integral membrane proteins that are present in almost every cell and in every kingdom of life. They are essential for the regulation of intracellular pH-value, Na^+^-concentration and cell volume. These secondary active transporters exchange sodium ions against protons via an alternating access mechanism, which is not understood in full detail. Na^+^/H^+^ antiporters show distinct species-specific transport characteristics and regulatory properties that correlate with respective physiological functions. Here we present the characterization of the Na^+^/H^+^ antiporter NhaA from *Salmonella enterica* serovar Thyphimurium LT2, the causing agent of food-born human gastroenteritis and typhoid like infections. The recombinant antiporter was functional *in vivo* and *in vitro*. Expression of its gene complemented the Na^+^-sensitive phenotype of an *E. coli* strain that lacks the main Na^+^/H^+^ antiporters. Purified to homogeneity, the antiporter was a dimer in solution as accurately determined by size-exclusion chromatography combined with multi-angle laser-light scattering and refractive index monitoring. The purified antiporter was fully capable of electrogenic Na^+^(Li^+^)/H^+^-antiport when reconstituted in proteoliposomes and assayed by solid-supported membrane-based electrophysiological measurements. Transport activity was inhibited by 2-aminoperimidine. The recorded negative currents were in agreement with a 1Na^+^(Li^+^)/2H^+^ stoichiometry. Transport activity was low at pH 7 and up-regulation above this pH value was accompanied by a nearly 10-fold decrease of K_m_^Na^ (16 mM at pH 8.5) supporting a competitive substrate binding mechanism. K^+^ does not affect Na^+^ affinity or transport of substrate cations, indicating that selectivity of the antiport arises from the substrate binding step. In contrast to homologous *E. coli* NhaA, transport activity remains high at pH values above 8.5. The antiporter from *S.* Typhimurium is a promising candidate for combined structural and functional studies to contribute to the elucidation of the mechanism of pH-dependent Na^+^/H^+^ antiporters and to provide insights in the molecular basis of species-specific growth and survival strategies.

## Introduction

Na^+^/H^+^ antiporter are integral membrane proteins that are crucial for control of intracellular pH, cellular Na^+^ concentration and cell volume in all biological kingdoms of life [1], [2]. They are secondary active transporters that exchange sodium ions against protons across the membrane. Eukaryotic, prokaryotic and archael Na^+^/H^+^ antiporters or exchangers were grouped in two clades of the cation-proton-antiporter (CPA) superfamily [1]–[3]. They share in general a common alternating access mechanism. Yet, in line with their broad range of physiological functions, they have distinct individual transport characteristics and regulatory properties. The human NHE isoforms, which belong to the CPA1 family, carry out neutral exchange with a 1Na^+^/1H^+^ stoichiometry [3]. They are crucial for numerous physiological processes and changes in their activities are implicated in the pathogenesis of several diseases as for instance essential hypertension, congenital secretory diarrhea, diabetes, epilepsy, and ischemia–reperfusion injury [4]. Na^+/^H^+^ antiporter are also important for ion homoeostasis of bacteria, which have to survive in rapidly changing or adverse environments [2]. Recently, deletion of the two major Na^+^/H^+^ antiporters NhaA and NhaB of *Yersinia pestis* showed that their function is indispensable for virulence of the causative agent of plague [5]. NhaA and NhaB are electrogenic transporters with stoichiometries of 1Na^+^/2H^+^ and 2Na^+^/3H^+^, respectively. The Na^+^/H^+^ antiporter NhaA from *Escherichia coli* (EcNhaA) is the best characterized member of the CPA2 family [2], [6]. NhaA is essential for growth of *E. coli* at alkaline pH-values in the presence of Na^+^- or Li^+^-ions [7], [8]. The transport is highly selective for Na^+^ and Li^+^ [9], [10]. The X-ray structure of EcNhaA revealed a new fold and provided first insights into the structural basis of an alternating access mechanism [11]–[13]. The position of key catalytic residues were identified and a potential Na^+^ binding site in the centre of the antiporter was suggested [11]. Binding as the determinant of selectivity was recently challenged by computational and experimental data which indicated that all alkali ions competitively bind to bacterial NhaA suggesting a promiscuous binding site with selectivity arising from a later step of the transport cycle [14]. A further interesting aspect is that Na^+^/H^+^ antiporters have characteristic individual pH-dependent activity profiles, which are important for their specific homoeostatic functions [6], [15]. EcNhaA transport activity drastically increases between pH 7.0 and pH 8.5 [7], [16]. A cytosolic pH-sensor responsible for pH-dependent regulation has been proposed and intensively studied for EcNhaA [17]. Electrophysiological characterization of pure, reconstituted transporter did confirm the pH profile [18], [19]. Yet, a recent study revealed that at asymmetrical pH conditions with a high pH at the Na^+^ uptake side, the transporter is highly active at low pH, even if the putative pH-sensor is exposed to acidic pH-values [20]. Based on this observation, a kinetic competition model was suggested in which Na^+^ and H^+^ compete for a common binding site [20], [21]. To date, the mechanism of pH-dependent transport is not understood in detail. Furthermore, species-specific differences in activity profile have been studied in few cases only, for instance for NhaA from *Helicobacter pylori* (HpNhaA) which was reported to be active in the pH-range between 6.0 and pH 8.5 [22]. Notably, transport activity of both EcNhaA and HpNhaA declines above pH 8.5 which was proposed to be caused by H^+^ substrate depletion at high alkaline pH-values [20]. Further characterization of species-specific properties of NhaA is important for a detailed understanding of the mechanism of pH-dependent transport and will contribute to the understanding of the molecular basis of species-specific growth and survival strategies.

To obtain insights in the pH-dependent Na^+^/H^+^ exchange of *Salmonella enterica*, we choose NhaA of *Salmonella enterica* serovar Typhimurium LT2 (*S.* Typhimurium), as this serovar is extensively characterized and the genome sequence is available [23]. *Salmonella enterica* species are Gram-negative, facultatively anaerobic bacteria of the family of *Enterobacteriaceae*. Several serovars can cause nontyphoidal foodborn human gastroenteritis with 94 million cases and 155,000 deaths estimated globally per year [24]. *Salmonella enteriditis nha*A was originally identified in a larger DNA fragment cloned by complementation [25]. That fragment harboured two open reading frames which encoded proteins homologous to the antiporter *nha*A and the gen regulator *nha*R, expression increased Na^+^/H^+^ antiport activity and also showed activity for Li^+^.

Here we show functionality of recombinant NhaA from *S.* Typhimurium *in vivo* and *in vitro* and describe species-specific properties, notably stable transport activity at alkaline pH-values. Electrophysiological measurements support the proposed competition mechanism and oppose the concept of a promiscuous cation binding site. The transporter is an attractive candidate to obtain further mechanistic insights in pH-dependent Na^+^/H^+^ antiport by combined structure/function analysis.

## Materials and Methods

### Production of recombinant STNhaA

The coding region for STNhaA (*nhaA*, STM0039) was amplified from genomic DNA of *S.* Typhimurium using the primer sequences CTTGGGATCCTTAAACATCTGCACCGA-TTTTTTAGCAGCG and CTGAGGAATTCGTTGCCGGCGCGTTCAAACGC, which include BamHI and EcoRI restriction sites, respectively. The PCR-fragment was inserted via these restriction sites into the expression plasmid pTTQ18A1 [26], a derivative of pTTQ18 [27], resulting in the expression construct pTTQ18A1-STNhaA-His_6_ which encodes for wild-type STNhaA with a TEV protease cleavable hexahistidine-tag fused to the carboxy-terminus (additional C-terminal residues FENLYFQGGRGGHHHHHH) and adds five amino acid residues NLGIL at the amino-terminus. For heterologous over-expression in *E. coli*, cells of strain NM554 [26] were transformed with pTTQ18A1-STNhaA-His_6_. Expression cultures were grown in LB medium at 37°C in shaking flasks. Protein production was induced at OD_600 nm_ = 0.5 with 0.5 mM isopropyl-β-D-thiogalactopyranoside for 3 h. Cells were harvested by centrifugation and membranes were prepared as previously described for EcNhaA [13] with minor modifications.

### IMAC purification of STNhaA

Membranes were solubilized in buffer (1% n-dodecyl-β-D-maltopyranoside (β-DDM), 50 mM HEPES pH 7.5, 100 mM NaCl), incubated for 1 h at 4°C and centrifuged at 4°C for 15 min at 310,000×g. The supernatant was loaded onto a 5 ml HisTrap HP (GE-Healthcare) column equilibrated with loading buffer (20 mM Tris/HCl pH 7.5, 500 mM NaCl, 5 mM imidazole and 0.03% β-DDM). The protein was eluted using 20 mM Na-Citrat pH 4.0, 100 mM NaCl, 0.03% DDM, with 500 mM imidazol. After desalting chromatography (HiTrap, GE-Healthcare), the protein was concentrated to 6 mg/ml by spin filtration.

### Blue-native-PAGE

IMAC purified STNhaA (7 µg) was incubated on ice for 45 min in presence of 1x sample buffer (Invitrogen, BN2003) and 3x cathode buffer (Invitrogen, BN2002). After centrifugation at 10,000×g for 5 min at 4°C, supernatant was loaded on a 4–16% BisTris NativePage gel (Invitrogen) and separated in Dark Blue Buffer (Invitrogen) for 2 h at 150 V.

### Size exclusion chromatography multi-angle laser-light scattering analysis

STNhaA solution (40 µl) was filtered using a 0.1 µm filter and subjected to two coupled Superdex 200 5/15 (GE-Healthcare) gel filtration columns equilibrated with size exclusion chromatography (SEC)-buffer (10 mM MES pH 6, 150 mM NaCl, 0.03% β-DDM). Separation was performed on an Äkta Micro System (GE-Healthcare) with in line monitoring of static light scattering (miniDAWN Treos, Wyatt Technology), differential refractive index (Optilab rEX, Wyatt Technology) and UV_280_ absorption. Data were processed with the ASTRA software package using the protein conjugate analysis tool (Wyatt Technology) with the calculated UV_280_ extinction coefficient (Expasy ProtParam tool [28]) of 57535 M^−1 ^cm^−1^ for STNhaA and a d*n*/d*c*_protein_ value of 0.185 ml/mg [29]. The d*n*/d*c* value for β-DDM in SEC buffer was determined experimentally by injecting different β-DDM concentrations into the differential refractive index detector and measuring the β-DDM concentration dependent change in refractive index.

### Semiautomated micro-culture growth complementation under salt stress

Production of functional STNhaA was probed with a growth complementation assay using the Na^+^/H^+^-antiporter deficient *E. coli* strain EP432 (*mel*BLid, Δ*nha*A1::*kan*, Δ*nha*B1::*cat*, Δ*lac*ZY, *thr*1 [30]. EP432 cells were transformed either with pTTQ18A1-STNhaA-His_6_ or with pTTQ18A1 vector. Pre-cultures in LBK were inoculated with a single colony. Aliquots of the pre-culture were diluted to an OD_600 nm_ of 0.02 in buffered (50 mM HEPES pH 7.5 or Tris/HCl pH 8.0) LB-medium (10 g/l bactotryptone, 5 g/l yeast extract) complemented with either 200 mM NaCl, 800 mM NaCl or 200 mM LiCl. 100 µl aliquots of the diluted suspension were applied to each well of a 96-well microtiterplate, which was sealed with an adhesive membrane (Breathe-Easy, Sigma-Aldrich). Growth was monitored every 10 min at 600 nm for up to 22 h using a microplate reader (Powerwave XS2, Biotek) at 37°C and under continues shaking. Assays were performed in triplicates in at least three independent experiments.

### Background expression of pTTQ18A1-STnhaA

Presence of STNhaA in non-induced cultures was determined with 5 ml cultures of EP432 *E. coli* cells transformed with pTTQ18A1-ST*nha*A in LB-medium containing 200 mM LiCl buffered at pH 8.0 for 22 h at 37°C. At different time points, 1 ml samples were taken, cells sedimented and resuspended in SDS sample buffer. Samples were normalized to 1 ml of culture with an OD_600_ = 0.8 and 20 µl were applied to 12% Tris/Glycin gels. After SDS-PAGE separation, samples were transferred to a polyvinylidene fluoride membrane and STNhaA was probed by Western immunoblotting with antibodies against the His-tag.

### Solid-supported-membrane (SSM) based electrophysiology

Purified STNhaA was reconstituted in proteoliposomes using the protocol described for EcNhaA [19]. SSM measurements were performed as described previously [31]. Briefly, 20 µl of proteoliposomes at a lipid concentration of ∼5 mg/ml were adsorbed to an octadecanethiol/phospholipid hybrid bilayer on a gold surface (sensor). Proteoliposomes were allowed to adsorb to the sensor for 60–90 min. Electrogenic transport was initiated by a rapid Na^+^ concentration jump. Solution exchange protocol: nonactivating solution (no Na^+^) 0.5 s; activating solution (with Na^+^) 0.5 s; nonactivating solution 0.5 s. Currents recorded throughout the measurements were amplified with a current amplifier set to a gain of 10^8^–10^9 ^V/A and low pass filtering set to 300–1000 Hz. To minimize Na^+^ concentration jump artefacts, Na^+^ was replaced by an equal amount on an inert cation (K^+^ or choline^+^) in the nonactivating solution. In addition, the ionic strength was kept high by adding K^+^ (or choline^+^) to a total cation concentration of 300 mM. The experiments were performed at room temperature.

The peak currents of each Na^+^-dependence (3 data sets) were normalized to the corresponding V_max_ values resulting from a hyperbolic fit to the individual data sets and then averaged. The peak currents of the pH-dependence (3 data sets) were approximated using a spline function and the individual data sets normalized to the maximal value of the corresponding spline function. Then, the normalized data sets were averaged. Details of the measurement and analysis procedure were previously published [20].

## Results

### NhaA from S. Typhimurium is functional *in vivo*

**Figure 1:**
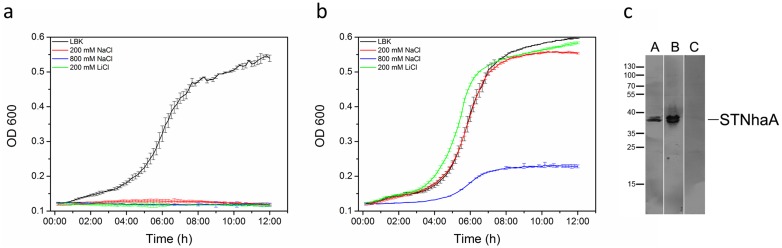
Expression of *S*. Typhimurium *nha*A complements salt-sensitive phenotype of Na^+^/H^+^ antiporter deficient *E. coli* strain EP432. Growth characteristics were monitored in microscale liquid growth complemention assays. Cells transformed with pTTQ18A1 (a) and with pTTQ18A1-STNhaA (b) were grown on LBK medium (67 mM KCl) as control. Salt-stress was induced with 200 mM NaCl, 200 mM LiCl, or 800 mM NaCl supplemented media buffered at pH 7.5. Triplicate samples were averaged and standard deviations for each trace ranged between 0.3 and 2.3%. Basal expression levels without chemical induction were used for the complementation assays. The leaky expression system resulted in substantial STNhaA production in cultures as used in (b). NhaA was detected by Western-immunoblot with anti-His-tag antibodies after SDS-PAGE separation of total cell extracts after 8 h (A) and 22 h (B) cultivation. As a control, cells were transformed with pTTQ18A1 and cultivated in LBK medium for 22 h (C).

Recombinant NhaA from *S.* Typhimurium was produced with a carboxy-terminal hexa-histidine tag by heterologous overexpression in *E. coli* (STNhaA hereafter). Functionality *in vivo* was analysed by growth complementation of the salt-sensitive Na^+^/H^+^ antiporter deficient *E. coli* strain EP432. This strain lacks the genes for NhaA and NhaB, does not grow on Na^+^- or Li^+^-supplemented selection media, and was previously used in physiological studies to probe functionality of Na^+^/H^+^ antiporter [6], [32], [33]. Growth complementation was performed with a semiautomated micro-culture growth assay [34] using media buffered at pH 7.5 (Fig. 1). Control cultures of cells transformed with the plasmid pTTQ18A1 grew on KCl-supplemented media and showed no growth upon addition of 200 mM NaCl or LiCl (Fig. 1a). Growth characteristics are similar to those previously described for shaking flask cultures [33] or micro-cultures of this strain [35] with stable growth over 2–3 hours. Complementation was analysed for EP432/ST*nhaA*-pTTQ18A1 cultures. As cells are less viable when overexpression is induced (data not shown), the basal expression of non-induced cultures was used. The presence of STNhaA in non-induced cells after 8 h and 22 h growth was confirmed by SDS-PAGE and consecutive Western-immunoblot probing the His-tag (Fig. 1c). Basal ST*nha*A expression fully conferred resistance to 200 mM NaCl or LiCl. It could ameliorate toxicity of high NaCl concentration (800 mM), though growth of these cultures is impaired. Taken together, STNhaA functionally complemented the salt-sensitive phenotype of the antiporter deficient *E. coli* strain under Na^+^ as well as Li^+^ stress at pH 7.5. Micro-cultures provide an efficient assay format which might be of interest for inhibitor screening.

### Dimeric STNhaA was purified to homogeneity

**Figure 2:**
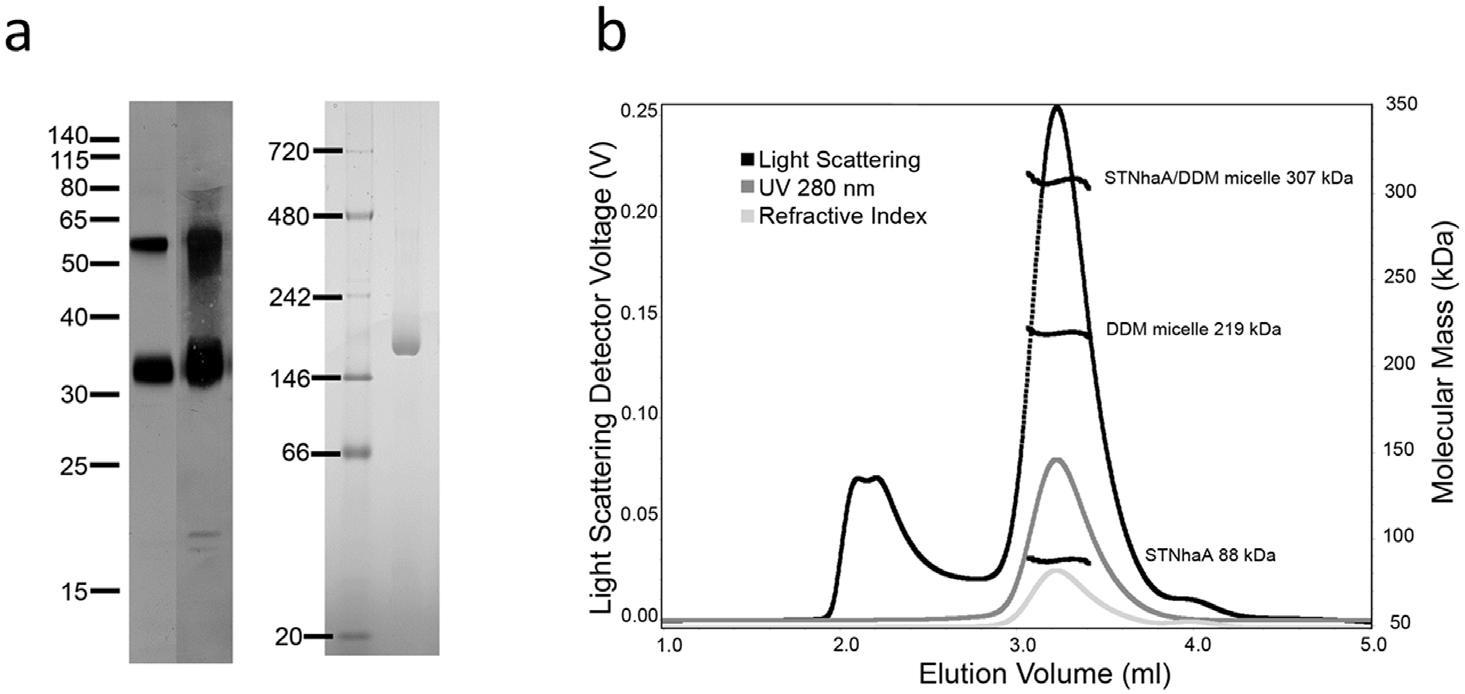
Purity and oligomeric state of STNhaA. (a) IMAC-purified STNhaA probed by SDS-PAGE analysis, Western-immunoblot and BN-PAGE. Shown is the silver-stained gel (12% Bis/Tris gel) after electrophoretic separation of 200 ng of STNhaA (left panel, left lane). Molecular masses of co-separated standard proteins are indicated in kDa. NhaA (33 kDa) and its SDS-resistant oligomer (53 kDa) were positively probed in Western-immunoblot with anti-His-tag antibodies (left panel, right lane). Coomassie-stained gel (4–16% Bis/Tris gel) after BN-PAGE analysis of 7 µg of IMAC purified STNhaA (right panel). The molecular masses of co-separated standard proteins are indicated on the left in kDa. (b) Chromatogram of SEC-MALLS analysis of β-DDM solubilized STNhaA. 80 µg of IMAC-purified STNhaA were applied to size-exclusion chromatography on a Superdex 200 column. The chromatogram shows detector readings of the 90 light scattering detector, the UV_280_ detector, and the refractive index detector in black, dark gray and light gray, respectively. The horizontal black lines indicate the calculated mass of STNhaA, detergent micelle, STNhaA/detergent complex and indicate a high degree of monodispersity throughout the elution peak.

STNhaA was purified from solubilized membranes by immobilized metal affinity chromatography (IMAC) with an average yield of 1.6 mg protein per litre of cell culture. The protein was highly pure as shown by SDS-PAGE analysis with silver-staining (Fig. 2a). It migrated with an apparent molecular mass of 33 kDa, faster as compared to the calculated mass (44.01 kDa). The higher electrophoretic mobility is often observed for integral membrane proteins which lack soluble domains and largely consist of transmembrane helices [36]. A second band at higher molecular mass (53 kDa) was present, most likely a dimeric association of the protein. The carboxy-terminal affinity-tag of the antiporter was intact as shown by Western-immunoblot with His-tag specific antibodies, which decorated both bands (Fig. 2a).

BN-PAGE analysis of purified STNhaA resulted in a single band at 176 kDa, suggesting an oligomeric state of native STNhaA (Fig. 2a, right panel). Purified STNhaA was stable and monodisperse in solution as documented by subsequent analytical size exclusion chromatography (SEC) (Fig. 2b). SEC analysis was combined with online multi-angle laser-light scattering and refractive index monitoring (SEC-MALLS). A mass of 88 kDa was determined for STNhaA in solution, in accurate agreement with the theoretical mass of the antiporter dimer. For analysis of the mass of the entire detergent/protein complex and the contribution of the detergent micelle, the d*n*/d*c* value of β-dodecyl-maltopyranoside (DDM) in SEC-buffer was experimentally determined to be 0.122 ml/g (Fig. S1). Comparison with previous values of 0.133 ml/g [37] and 0.143 ml/g [38] for DDM emphasizes that d*n*/d*c* values for detergents vary with buffer conditions [38]. Based on the specific d*n*/d*c* value, the STNhaA/DDM complex had a mass of 307 kDa for the entire complex, of which a substantial mass of 219 kDa were contributed by the associated detergent micelle. For comparison, a pure β-DDM micelle has a mass of 72 kDa [37]. A similar contribution of 233 kDa DDM was reported for the protein/detergent complex of the dimeric metal transporter YiiP [39], [40]. The pure, monodisperse preparation of STNhaA suggests this transporter as a promising candidate for structural and functional characterization. Antibody-mediated crystallization may be a good strategy to accommodate the large detergent micelle [41].

### Electrophysiological characterization of Na^+^(Li^+^)/H^+^ transport of reconstituted STNhaA

**Figure 3:**
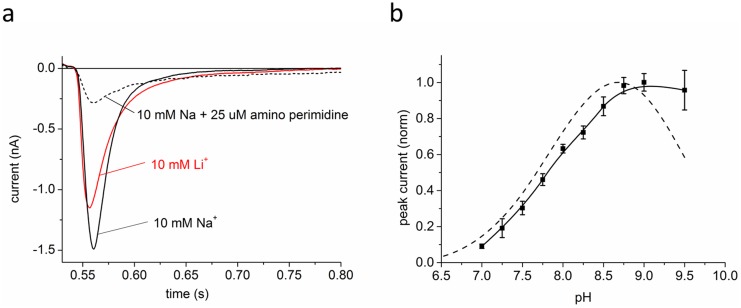
Kinetic characterization of STNhaA. (a) Transient electrical currents generated by purified STNhaA reconstituted in proteoliposomes using SSM-based electrophysiology after 10 mM Na^+^ and Li^+^ concentration jumps at pH 8.5. The buffers contained 25 mM Tris/25 mM MOPS/25 mM HEPES pH 8.5 (Tris), 290 mM KCl and 0.1 mM DTT. In addition, the activating and the nonactivating solutions contained 10 mM NaCl and KCl, respectively. The dashed line shows inhibition of the NaCl concentration jump signal with 25 µM 2-aminoperimidine. (b) The pH dependence of STNhaA peak currents after a 100 mM Na^+^ concentration jump. The buffers contained 25 mM Tris/25 mM MOPS/25 mM HEPES at pH values indicated (HCl or Tris), 200 mM KCl and 0.1 mM DTT. In addition, the activating and the non-activating solutions contained 100 mM NaCl and KCl, respectively. The dashed line represents the corresponding pH dependence of EcNhaA [20], [21].

For functional characterization, purified STNhaA was reconstituted in liposomes and transport activity was assayed with direct current measurements using SSM-based electrophysiology. Transient electrical currents generated by STNhaA were observed after imposing concentration jumps of Na^+^ or Li^+^ on proteoliposomes. Typical currents recorded upon a 10 mM Na^+^ (Li^+^) concentration jump are shown in Fig. 3a. The peak currents correspond to stationary turnover of the Na^+^/H^+^ exchanger and can be used to characterize its transport activity [19]. According to the transport stoichiometry of 1Na^+^/2H^+^, negative currents corresponding to the displacement of positive charge out of the proteoliposomes were recorded upon a Na^+^ concentration jump. Clearly, Na^+^ and Li^+^ were both transported by STNhaA. Li^+^ had a slightly lower turnover than Na^+^. The amiloride-analogue 2-aminoperimidine (2-AP) was described as specific inhibitor of bacterial NhaA Na^+^/H^+^ antiporters [42]. In agreement, the transient current generated by a 10 mM Na^+^ concentration jump was nearly completely suppressed by addition of 25 µM 2-AP (Fig. 3a).

### STNhaA transport and affinity for Na^+^ are pH dependent

**Figure 4:**
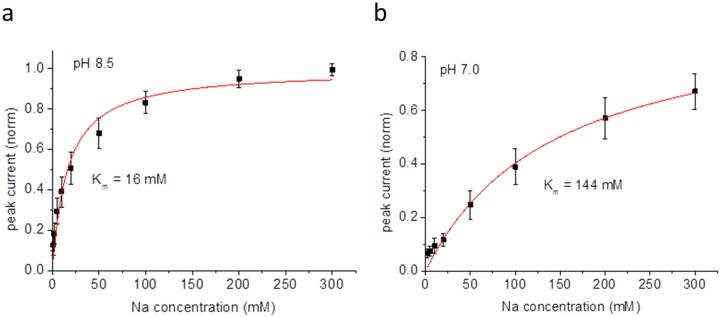
Na^+^ concentration dependence of STNhaA peak currents after a Na^+^ concentration jump of the indicated concentration at pH 8.5 and pH 7.0. Experiments were performed with reconstituted STNhaA as in Fig. 3. The buffers contained 25 mM Tris/25 mM MOPS/25 mM Hepes pH 8.5 or 7.0 (adjusted with HCl at pH 7.0 or Tris at pH 8.5). In addition, the activating solution contained×mM of NaCl with (300-x) mM background of KCl, the non-activating solution only 300 mM KCl. Currents were normalized and averaged from 3 different data sets as described in Materials and Methods. The solid line is a fit to the data using hyperbolic concentration dependence. The respective K_m_ values for Na^+^ are indicated in the figure.

The pH-dependence of STNhaA transport activity was characterized with currents recorded after concentration jumps of 10 mM Na^+^ performed at pH-values ranging from pH 7.0 to pH 9.5 (Fig. 3b). Currents were small at pH 7 and increased in a sigmoidal manner with rising pH-values reaching 50% at ∼pH 7.8 and a maximum at pH 9.0. The transport activity remained high at pH 9.5. The pH-profile was slightly shifted as compared to NhaA from *Escherichia coli* (EcNhaA, Fig. 3b) and, notably, transport activity of that transporter steadily declined above pH 8.5 [19]. A competitive mechanism in which Na^+^ and H^+^ compete for a common binding site has been suggested for down regulation of transport activity at low pH [20]. In order to extend this hypothesis on STNhaA, the binding constants for Na^+^ at pH 8.5 and 7.0 were determined (Fig. 4). Indeed, the K_m_ for Na^+^ 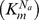 increased from 16 mM at pH 8.5 to 144 mM at pH 7.0, in line with a competitive mechanism.

### Selectivity of cation binding and transport

**Figure 5:**
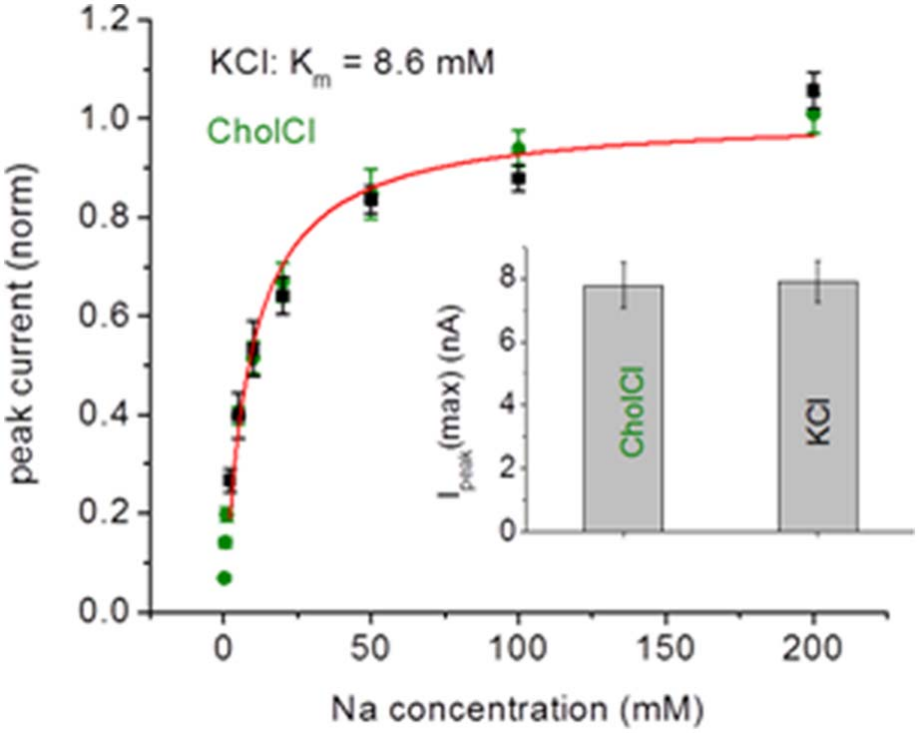
Probing competitive K^+^ inhibition of the peak currents generated by STNhaA after a Na^+^ concentration jump. Experiments were performed with reconstituted STNhaA as in Fig. 3. The buffers contained 25/25 mM MOPS/25 mM HEPES pH 8.5 (adjusted with Tris). In addition, the activating solution contained×mM of NaCl with (300-x) mM background of KCl (black squares) or CholCl (green circles), the non-activating solution only 300 mM KCl or CholCl. The red line shows a fit to the data obtained with KCl background. Three independent measurements with KCl and CholCl were perfomed, normalized and averaged as described in Experimental Procedures. The saturating currents determined before normalization for KCl and CholCl are indicated.

Promiscuous ion binding in bacterial NhaA was recently suggested, as computational data showed that all alkali ions bind to the active site of EcNhaA without particular preference and that they inhibit NhaA activity in everted membrane vesicles [14]. This is in contrast to the high selectivity for Na^+^ and Li^+^ shown for that transporter [9], [10], [19]. To challenge the hypothesis for bacterial NhaA, we analysed whether elevated concentrations of K^+^ impair transport function of STNhaA. For this purpose, Na^+^-dependent electrical currents were measured with KCl and choline chloride (CholCl) background adjusted to a total salt concentration of 300 mM using SSM-based electrophysiology. Normalised and averaged data are presented in Fig. 5. The K_m_ for Na^+^ determined with KCl background (8.6 mM) and CholCl background (8.0 mM) were identical within the experimental error as were the saturation values for the peak currents Ipeak(max) determined before normalization (Fig. 5 see insert). These measurements correspond to a 30-fold excess of KCl or CholCl at half-saturating Na^+^ concentrations without a detectable effect of K^+^ on the K_m_ for Na^+^. We also performed experiments in the absence of a salt background at half-saturating Li^+^ concentrations (3 mM) with and without 100 mM KCl (data not shown). No inhibition of Li^+^ transport was observed. In conclusion, neither STNhaA transport activity nor affinity for Na^+^ were affected by KCl, demonstrating that substrate selectivity of STNhaA already arises at the ion binding step.

## Discussion

The Na^+^/H^+^ antiporter NhaA from *S.* Typhimurium was produced in functional form by heterologous expression in *E. coli*. ST*nha*A expression efficiently complements the Na^+^ and Li^+^ sensitive growth phenotype of the Na^+^/H^+^ antiporter (Δ*nha*A Δ*nha*B) deficient *E. coli* strain EP432. This *in vivo* functionality is in agreement with the initial characterization of a larger DNA fragment containing *nha*A and the gene regulator *nha*R [25]. Overproduction and purification of STNhaA resulted in milligram amounts of pure and stable protein per litre of expression culture ideal for biochemical and biophysical characterization. BN-PAGE separation of purified STNhaA showed a homogenous oligomeric state with an apparent mass of 176 kDa. A factor of 1.8 to be applied to the theoretical mass of integral membrane transport proteins was suggested to correct for bound detergent, lipid and Coomassie-Blue molecules in BN-PAGE [43]. Based on this correction, a dimeric association of STNhaA in BN-PAGE can be assumed, as the experimental result is close to the corrected apparent mass of a dimer (158 kDa). One should note that PN-PAGE mobility can be substantially influenced by detergent, lipid and dye molecules, as recently shown for the mitochondrial ADP/ATP carrier for which the apparent mass varied between 60 and 130 kDa depending on solubilisation conditions [44]. Alternatively, SEC-MALLS permits a precise analysis of the oligomeric state of membrane proteins in solution, as protein and detergent contributions can be discriminated for a monodisperse protein detergent complex that elutes as a single peak in size exclusion chromatography. The STNhaA homodimer in solution was accurately identified with a mass of 88 kDa by SEC-MALLS. Bacterial Na^+^/H^+^-antiporters appear to be physiological dimers. EcNhaA is dimeric in native as well as artificial membrane environments shown by cryo-electron microscopy of two-dimensional crystals [45] and by genetic and biochemical methods [46]. Cross-linking of monomers at the dimer interface changed the pH profile of EcNhaA transport [46]. The functional role of the dimeric association is not entirely clear, as monomers of EcNhaA are fully functional. Dimerization may be beneficial under extreme stress conditions at alkaline pH in the presence of Na^+^ or Li^+^ [33].

Purified STNhaA is fully capable of electrogenic Na^+^/H^+^ and Li^+^/H^+^ exchange *in vitro* when reconstituted in proteoliposomes and assayed by SSM-based electrophysiology. The characterization showed that 1) the negative currents are in agreement with a 1Na^+^/2H^+^ stoichiometry, 2) the 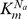 is 16 mM at pH 8.5 when the transport rate is close to maximum, 3) transporter activity is low at pH 7 and steadily increases with rising pH, and 4) acidic down-regulation is accompanied by a nearly 10-fold increase of 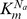 (144 mM at pH 7.0). The kinetic properties compare overall well with those of EcNhaA, for which 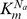 values of 11 mM and 178 mM were reported at pH 8.5 and pH 7, respectively [20]. Importantly, the distinct change in Na^+^ affinity of STNhaA supports the hypothesis that a competitive mechanism is responsible for acidic pH regulation of bacterial NhaA Na^+^/H^+^ antiporters, in which Na^+^ and H^+^ compete for the same binding site [20].

The cation/proton antiport activity of bacterial NhaA is highly efficient for Na^+^ and Li^+^. STNhaA as well as EcNhaA [9], [10] activity supports growth of antiporter deficient *E. coli* cells at high Li^+^ concentrations or at alkaline pH in the presence of Na^+^. Cation specificity was emphasised by characterization of transport kinetics of EcNhaA with SSM-based electrophysiology. Whereas Na^+^ and Li^+^ generated similar peak currents at pH 8.0 and were transported with similar turnover rates, neither K^+^ or Rb^+^ elicited current signals [19]. In line with these data, STNhaA cation/proton antiport activity was shown for Na^+^ and Li^+^ with similar peak currents. A conflicting study suggested, based on computational analysis of ion binding and transport studies on isolated membranes, that all alkali ions compete for substrate binding and that for instance K^+^ or Rb^+^ binding inhibits NhaA transport activity [14]. However, the SSM studies with purified reconstituted STNhaA clearly showed that K^+^ is not competing with Na^+^ binding or Na^+^/H^+^ transport activity, clearly indicating that selectivity is already conveyed at the binding step.

The overall agreement in kinetic properties of STNhaA and EcNhaA coincides with the amino acid sequence identity of 92%. Residues Asp163 and Asp164, which are essential for ion transport in EcNhaA [47], and other residues of structural and mechanistic importance, for instance Thr132, Asp133, Lys300 [48] are fully conserved. However, the two transporters differ in one aspect of function despite their high sequence identity. While EcNhaA activity irreversibly decreases above pH 8.5 [19], the transporter from *S.* Typhimurium maintains its maximum activity in electrophysiological measurements at least up to pH 9.5. Alkaline down regulation in EcNhaA has been explained by substrate H^+^ depletion [20], a mechanism which is in the physiological transport mode of NhaA a function of periplasmic pH. For STNhaA, high activity at pH 9.5 indicates that the H^+^ transport step is fast, permitting the transporter to operate without rate limitation even at low H^+^ concentration. It may also reflect a higher stability of the protein at alkaline pH. Further experiments need to address structure/function relationships that lead to the species-specific pH profile and may also explore the functional role of STNhaA for growth and survival of *Salmonella* in adverse environments. Further species-specific properties were recently described for NhaA from *Helicobacter pylori*, for which the pH profile is shifted by 1.2 pH units to a more acidic range as compared to STNhaA [49]. In addition, the Na^+^/H^+^ antiporter NapA from *Thermus thermophilus* shows down-regulation at pH 6 only for Na^+^/H^+^ antiport, whereas Li^+^/H^+^ activity remains high at acidic pH [35]. A detailed structural and functional characterization of different bacterial Na^+^/H^+^ antiporters is required for a full understanding of species-specific activities and will contribute to elucidation of the pH-dependent Na^+^/H^+^ transport mechanism in general. The X-ray structure of EcNhaA was obtained at low pH in inward-open conformation [11] and conformational changes upon pH activation are debated [17]. The structure of the Na^+/^H^+^ antiporter NapA from *Thermus thermophilus* displays an outward-open conformation [50] but also lacks a bound sodium ion. The good biochemical properties and the sustained high activity of STNhaA at pronounced alkaline pH might be beneficial for crystallization of the protein in an active conformation.

## Supporting Information

Figure S1 **Determination of the d** ***n*** **/d** ***c*** **value for β-DDM in SEC buffer.**
(PDF)
