# Supplementary material for: Molecular Characterization of the Na+/H+-Antiporter NhaA from Salmonella Typhimurium
Source: PLoS One. 2014 Jul 10;9(7):e101575. doi: 10.1371/journal.pone.0101575 (PMC4092016; doi:10.1371/journal.pone.0101575)
Supplement: Figure S1 — Determination of the d n /d c value for β-DDM in SEC buffer. (PDF) [file pone.0101575.s001.pdf]

Molecular characterization of the Na^+/H^+-antiporter NhaA from *Salmonella* Typhimurium

Christopher J. Lentes[1,2], Syed H. Mir [1,3], Marc Boehm[4], Constanta Ganea[5], Klaus Fendler[6], Carola Hunte[1]

[1] Institute for Biochemistry and Molecular Biology, ZBMZ, BIOSS Centre for Biological Signalling Studies, University of Freiburg, Freiburg, Germany;[2]Faculty of Biology, University of Freiburg, Freiburg, Germany; [3] Dept. Clinical Biochemistry, University of Kashmir, Srinagar, India; [4] Dept. Molecular Membrane Biology, Max Planck Institute of Biophysics, Frankfurt/M., Germany; [5]Biophysical Department, Faculty of Medicine, Carol Davila University of Medicine and Pharmacy, Bucharest, Romania; [6] Dept. Biophysical Chemistry, Max Planck Institute of Biophysics, Frankfurt/M., Germany

Correspondence to Carola Hunte: Institute for Biochemistry and Molecular Biology, ZBMZ, BIOSS Centre for Biological Signalling Studies, Stefan-Meier-Strasse 17, University of Freiburg, 79104 Freiburg, Germany, E-mail:carola.hunte@biochemie.uni-freiburg.de

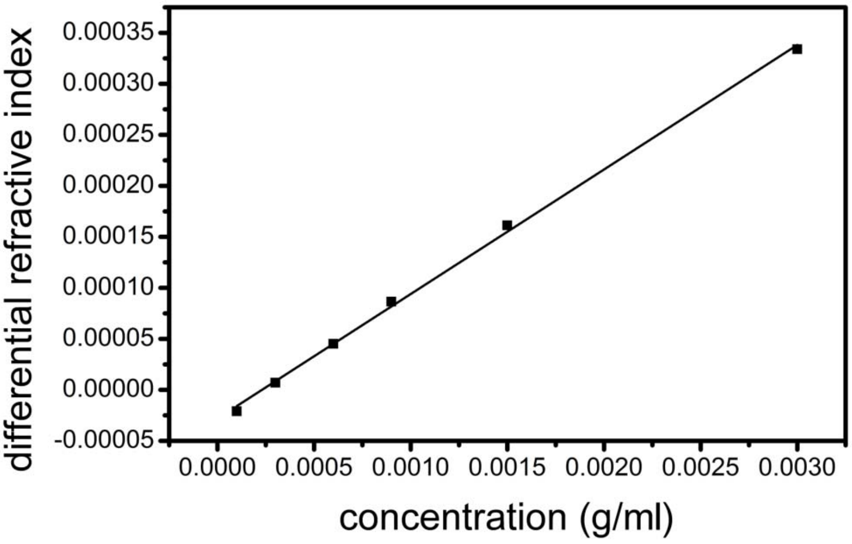

Figure S1. Determination of the d*n*/d*c* value for ß-DDM in SEC buffer. DDM concentration is given in g/ml. The dn/dc for ß-DDM was determined to be 0.122 mL/g.
